# The Many Faces of Hypusinated eIF5A: Cell Context-Specific Effects of the Hypusine Circuit and Implications for Human Health

**DOI:** 10.3390/ijms25158171

**Published:** 2024-07-26

**Authors:** Shima Nakanishi, John L. Cleveland

**Affiliations:** Department of Tumor Microenvironment & Metastasis, Moffitt Cancer Center, 12902 Magnolia Drive, Tampa, FL 33612, USA; john.cleveland@moffitt.org

**Keywords:** hypusine, eIF5A, DHPS, DOHH, polyamine, spermidine, cancer, metabolism, aging, immune senescence

## Abstract

The unique amino acid hypusine [N^ε^-(4-amino-2-hydroxybutyl)lysine] is exclusively formed on the translational regulator eukaryotic initiation factor 5A (eIF5A) via a process coined hypusination. Hypusination is mediated by two enzymes, deoxyhypusine synthase (DHPS) and deoxyhypusine hydroxylase (DOHH), and hypusinated eIF5A (eIF5A^Hyp^) promotes translation elongation by alleviating ribosome pauses at amino acid motifs that cause structural constraints, and it also facilitates translation initiation and termination. Accordingly, eIF5A^Hyp^ has diverse biological functions that rely on translational control of its targets. Homozygous deletion of *Eif5a*, *Dhps*, or *Dohh* in mice leads to embryonic lethality, and heterozygous germline variants in *EIF5A* and biallelic variants in *DHPS* and *DOHH* are associated with rare inherited neurodevelopmental disorders, underscoring the importance of the hypusine circuit for embryonic and neuronal development. Given the pleiotropic effects of eIF5A^Hyp^, a detailed understanding of the cell context-specific intrinsic roles of eIF5A^Hyp^ and of the chronic versus acute effects of eIF5A^Hyp^ inhibition is necessary to develop future strategies for eIF5A^Hyp^-targeted therapy to treat various human health problems. Here, we review the most recent studies documenting the intrinsic roles of eIF5A^Hyp^ in different tissues/cell types under normal or pathophysiological conditions and discuss these unique aspects of eIF5A^Hyp^-dependent translational control.

## 1. Introduction

**Figure 1:**
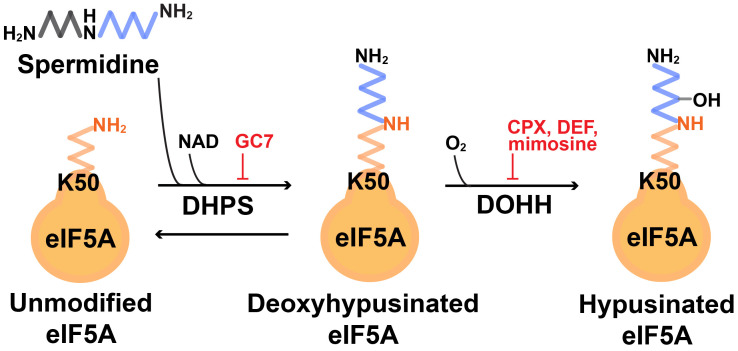
Schematic of the spermidine–hypusine circuit. Hypusination of eIF5A is mediated by the two enzymes, DHPS and DOHH, which can be inhibited by the DHPS inhibitor GC7, or the DOHH inhibitors CPX, DEF, or mimosine. Spermidine is the sole substrate for hypusination of eIF5A. CPX—ciclopirox; DEF—deferiprone; DHPS—deoxyhypusine synthase; DOHH—deoxyhypusine hydroxylase; eIF5A—eukaryotic translation initiation factor 5A; GC7—N1-guanyl-1, 7-diaminoheptane.

An unusual amino acid, hypusine [*N*^ε^ -(4-amino-2-hydroxybutyl)lysine] is a derivative of lysine and is covalently linked to the eukaryotic translation initiation factor 5A (eIF5A) [1]. The process of hypusine formation on eIF5A (coined hypusination) involves two enzymatic steps (Figure 1). First, the 4-aminobutyl moiety of the polyamine spermidine is transferred to the epsilon amino group of a specific eIF5A lysine residue (lysine-50 in human eIF5A) by deoxyhypusine synthase (DHPS) to form the intermediate deoxyhypusine-eIF5A. Secondly, deoxyhypusine is hydroxylated by deoxyhypusine hydroxylase (DOHH) to generate hypusinated eIF5A (eIF5A^Hyp^, a mature form of eIF5A) [1,2]. In humans, two isoforms of eIF5A, eIF5A1 (also called eIF5A) and eIF5A2 that has 84% identity to eIF5A1 [3], are the only proteins that undergo hypusination. eIF5A is ubiquitously expressed in most tissue types, whereas eIF5A2 is expressed in select tissues such as brain and testis [4].

eIF5A was originally identified as a translation initiation factor, as it promotes methionyl-puromycin synthesis in cell-free systems [5], and later was shown to also function in translation elongation and termination [6,7]. Since Kang and Hershey first reported that eIF5A may not be an absolute requirement for general translation, many studies, including ours, have shown that eIF5A is required for the translation of select mRNAs. Several independent well-designed mechanistic studies have demonstrated that eIF5A and its bacterial ortholog, EF-P (translation elongation factor P), are required for alleviating ribosome stalling at polyproline (>PPP) stretches and other proline-containing sequences during translation elongation [7,8,9,10]. Indeed, the pyrrolidine ring of proline confers structural constraints on amino acid positioning during peptidyl transfer, and the hypusine side chain of eIF5A is predicted to stabilize the binding of the peptidyl tRNA to the 80S ribosome and promote peptide bond formation [9,11]. Further, a study by Pelechano and Alepuz revealed that eIF5A-dependent ribosome pauses are found at more than 200 tripeptide motifs, including proline, glycine, and charged amino acid codons, and at termination sites in the yeast *S. cerevisiae* [12]. These newly identified tripeptides may also cause ribosome stalls in mammals in the absence of eIF5A, which may explain why depletion of eIF5A affects expression of proteins that do not possess the polyproline stretches. Furthermore, the *MYC* gene encodes five sites with such tripeptides having the diproline (Pro-Pro) motif, and mutation of all five sites can restore MYC protein levels in DHPS-depleted HCT116 colorectal cancer cells. In contrast, each individual mutation was not able to restore the protein levels [13], indicating that the number of such pausing sites may also determine the overall efficiency of MYC protein synthesis.

eIF5A, DHPS, DOHH, and hypusination of eIF5A are highly conserved in all eukaryotes. Germline homozygous deletion of any of these three genes leads to embryonic lethality in mice [14,15,16]. More recently, genetic studies using whole exome sequencing have revealed that rare genetic disorders in humans are linked to germline variants found in *EIF5A*, *DHPS*, and *DOHH* [17,18,19]. Firstly, heterozygous variants in *EIF5A* cause an autosomal dominant disorder, Faundes–Banka syndrome, which results in craniofacial neurodevelopmental malformations [17]. Secondly, biallelic variants of *DHPS* are associated with a rare inherited neurodevelopmental disorder [18], where all five affected individuals share a recurrent missense variant in trans with a second variant, resulting in mutant DHPS that has reduced enzyme activity and compromised hypusination of eIF5A. Biallelic variants in *DOHH* are also associated with a neurodevelopmental disorder and fibroblasts derived from these individuals have decreased DOHH enzyme activity, the accumulation of the intermediate deoxyhypusine eIF5A, and the consequent reduction of eIF5A^Hyp^ [19]. The phenotypes of these affected individuals include developmental delays, seizures, intellectual disability, microcephaly, and facial dysmorphisms, underscoring the essential roles of the hypusine circuit in neurodevelopment.

Although the effects of eIF5A^Hyp^ loss on overall global protein synthesis is rather modest, the lethal effects of germline deletion of *Eif5a*, *Dhps*, and *Dohh* on embryogenesis indicate that eIF5A^Hyp^ controls the translation of proteins essential for development. Recent advances in technologies and the availability of various genetic tools have made it possible to define the mechanisms by which eIF5A^Hyp^ contributes to distinct tissue and cell specific processes. Importantly, in addition to conventional knockout mice and mice carrying mutations in these genes, conditional knockout mice targeting *Dhps*, *Dohh, Eif5a*, and *Eif5a2* genes have been generated and are available to the public (Table 1), allowing one to assess the intrinsic roles of the hypusine circuit in different tissues and cell types under normal and disease conditions. Here, we review recent studies that have demonstrated cell context-specific roles of eIF5A^Hyp^ and discuss the many aspects of translational control by eIF5A^Hyp^ under normal and pathophysiological conditions.

## 2. Tissue/Cell Specific Roles of eIF5A^Hyp^

### 2.1. Gastrointestinal Tissues

#### 2.1.1. Intestinal Epithelium Cells (IECs)

Inflammatory bowel disease (IBD), including Crohn’s disease (CD) and ulcerative colitis (UC), is increasing worldwide and, furthermore, chronic inflammation is also associated with colitis linked to carcinoma. Patients with IBD show reduced levels of DHPS and eIF5A^Hyp^ in their colon, and mice having a specific deletion of *Dhps* in IECs develop chronic inflammation, are highly susceptible to dextran sulfate sodium (DSS), a widely used chemical colitogen, and are prone to develop more tumors following treatment with carcinogens [22]. eIF5A^Hyp^ translational targets identified by proteomics analysis of IECs isolated from mice lacking *Dhps* include enzymes involved in aldehyde detoxification, including glutathione S-transferases (GSTs) that catalyze the conjugation of aldehyde to glutathione (GSTA4, GSTM3, GSTM2, GSTP1, GSTO1, and GSTM1) and aldehyde dehydrogenases (ALDHs) that catalyze oxidation of aldehydes to carboxylates (AL1A7, AL1B1, and ALDH2) (Table 2). Notably, all these targets possess at least one of the previously reported mRNA sequence motifs of eIF5A^Hyp^-translational dependent transcripts, including AAAUGU [23] or diproline/diglycine [6,9] motifs. In this context, eIF5A^Hyp^ activity prevents chronic inflammation and carcinogenesis, suggesting a tumor suppressive role of eIF5A^Hyp^.

#### 2.1.2. Pancreas and β Cells

β cells in the pancreatic islet respond to changes in blood glucose levels by synthesis and secretion of insulin, which regulates blood glucose levels. Diabetes is a disorder of glucose homeostasis caused by the dysfunction or destruction of islet β cells. Type 1 diabetes (T1D) results from the autoimmune destruction of islet β cells (also see immune cells, Section 2.3. in this review), whereas Type 2 diabetes (T2D) occurs when insulin production fails to meet the demand.

Proliferation of β cells is normally low but can increase under pathophysiological conditions (e.g., insulin resistance). A study investigating the onset of facultative β cell mass expansion during obesity/insulin resistance using an inducible β cell-specific *Dhps* knockout mouse demonstrated that DHPS activity is increased in islets in response to a high-fat diet (HFD) and facilitates the induction of β cell proliferation and the maintenance of normal glucose homeostasis after HFD feeding [20]. Notably, β cell proliferation observed following HFD feeding is impaired in the *Dhps* knockout mice and mechanistically this defect is linked to reduced levels of cyclin D2 that is necessary for adaptive β cell growth, consequently leading to glucose intolerance.

#### 2.1.3. Pancreatic Ductal Adenocarcinoma (PDAC) Pathogenesis

PDAC is one of the most lethal cancers, with a very poor overall survival rate (<5% for metastatic PDAC), largely due to the late diagnosis. eIF5A, eIF5A2, and eIF5A^Hyp^ are all elevated in human PDAC tissues and in premalignant pancreatic intraepithelial tissues isolated from Pdx1-*Cre*;LSL-*Kras^G12D^* mice [24]. Detailed studies have shown that depletion of eIF5A in PDAC cells impairs cell growth ex vivo and orthotopic tumor growth in vivo, while increased expression of eIF5A promotes cell proliferation and tumor formation. Both DHPS and DOHH inhibitors, N1-guanyl-1,7-diaminoheptane (GC7) and ciclopirox (CPX), respectively (Figure 1), suppress PDAC growth. Further, PDAC cell growth is controlled by eIF5A^Hyp^ via translational control of the nonreceptor tyrosine kinase PEAK1, and PEAK1 overexpression rescues proliferation of cells having depleted eIF5A or eIF5A2. Moreover, the inhibition of eIF5A^Hyp^ by CPX treatment increases the sensitivity of PDAC to gemcitabine, the first-line chemotherapy for PDAC in both drug-resistant (PANC1) and drug-sensitive PDAC cells, suggesting the hypusine-PEAK1 axis as a potential therapeutic target. Finally, genetic depletion or pharmacological inhibition of eIF5A^Hyp^ impairs PDAC cell migration, invasion, and metastasis ex vivo and in vivo, indicating a role of eIF5A^Hyp^ in PDAC metastasis [25]. A proteomic analysis of human PDAC-derived cells (779E) with or without eIF5A depletion revealed that eIF5A^Hyp^ translationally regulates RhoA/ROCK2 signaling that controls actin/myosin mediated-cell spreading and migration.

### 2.2. Breast Cancer

Image-based high-throughput siRNA screens in MCF-7 human breast cancer cells expressing the autophagosome marker, GFP-LC3B, revealed eIF5A as a regulator of autophagy [26]. Indeed, eIF5A depletion results in the reduced lipidation of LC3B and its paralogs GABARAP and disrupts autophagosome formation. The lipidation of LC3 and GABARAP requires sequential processes involving the E1-like ATG7, the E2-like ATG3, and the E3-like ATG12-ATG5-ATG16L1 complex. Notably, proteomics analysis of eIF5A depleted cells revealed that ATG3 is an eIF5A-translational target. ATG3 directly regulates ATG8 family proteins, and the silencing of ATG3 reduces LC3B lipidation, phenocopying the effects of eIF5A knockdown, while ATG3 overexpression in eIF5A depleted cells rescues defects in LC3B lipidation.

### 2.3. Immune Cells

Translational control by eIF5A^Hyp^ is required in various immune cells to maintain cell lineage fidelity and further coordinate immune responses. The roles of the polyamine–hypusine circuit in controlling the immune system has been intensively studied and eIF5A^Hyp^ has been shown to control many aspects of immune cell fate and functions, in both physiological and pathophysiological states. Importantly, these findings support the hypusine circuit as a potential therapeutic target in disease conditions where immune cell functions are compromised or overactive.

#### 2.3.1. B Cells and B Cell Aging

B cells are central mediators of adaptive humoral immunity and play key roles in protecting against pathogens by producing antigen-specific immunoglobulin (Ig). Thus, reduced B cell function leads to increased risk of infections and poor vaccination efficiency. Antibody responses against pathogens are reduced in the elderly, making them particularly vulnerable to various infections.

Age-related reductions in autophagy occur in many organisms and the induction of autophagy by the polyamine spermidine promotes longevity in human T lymphocytes and cultured human peripheral blood mononuclear cells (PBMCs), and in yeast, *Drosophila*, and *C. elegans* [34]. Notably, the age-induced reductions of spermidine that occur in many organisms [35] hold true for B cells. Specifically, spermidine levels are significantly reduced in B cells in old mice, resulting in reduced levels of eIF5A^Hyp^, and inefficient translation of the master autophagy and lysosomal transcription factor TFEB and subsequent reductions in autophagic flux [27]. Interestingly, TFEB was the only autophagy-related protein identified by proteomics analysis. Specifically, TFEB is reduced in primary B cells treated with the DHPS inhibitor GC7, yet ATG3, which is regulated by eIF5A^Hyp^ in breast cancer cells [26] (see Section 2.2), is not affected in this context; thus, the translational control of autophagy regulators by eIF5A^Hyp^ is context dependent. Moreover, spermidine supplementation increases levels of eIF5A^Hyp^ and TFEB and improves B cell responses in old mice, indicating that the age-induced decline of spermidine is linked to autophagy-mediated immune senescence via translational control of eIF5A^Hyp^.

#### 2.3.2. B Cell Malignancies

Lymphomas are one of the most common cancers, and diffuse large B-cell lymphoma (DLBCL) is the most prevalent lymphoma subtype. MYC, a master transcriptional regulator of cancer cell growth (mass) and metabolism, is overexpressed by chromosomal translocations or other means in B-cell lymphomas. Indeed, 10% of DLBCL, 90% of Burkitt lymphomas (BL), 100% of double/triple-hit lymphomas, and 45% of high-grade B-cell lymphoma not otherwise specified (HGBL NOS) carry a *MYC* rearrangement [36,37,38]. *MYC* rearrangements are associated with a poor prognosis in DLBCL patients treated with R-CHOP [39]. We have shown that MYC coordinately induces the transcription of enzymes that direct the polyamine–hypusine circuit and, accordingly, levels of eIF5A^Hyp^ are significantly elevated in both human and mouse B-cell lymphoma with MYC involvement [28]. Importantly, both genetic and pharmacological studies have established that the polyamine–hypusine circuit is essential for development and maintenance of MYC-driven lymphoma. Specifically, (i) the loss of eIF5A hypusination by deletion of *Dhps* specifically in B cells abolishes malignant transformation of the B cells of Eμ-*Myc* transgenic mice (Eμ-*Myc*;CD19-*Cre*;Dhps*^fl/fl^*), and (ii) pharmacological inhibition, knockdown or knockout of *Dhps*, or knockdown of *Eif5a* suppresses the growth and survival of extant lymphoma both ex vivo and in vivo. Notably, RNA-seq, ribosome profiling, and proteomics analyses revealed that efficient translation of select targets is dependent upon eIF5A^Hyp^, including known oncogenes and regulators of G1-S phase cell cycle progression and DNA replication (POLD1, E2F1, E2F2, Cyclin D3, PIM3) (Table 2), indicating eIF5A^Hyp^ controls MYC-driven proliferation in premalignant B cells [28]. Interestingly, although TFEB is an eIF5A^Hyp^ translation target in normal B cells (see Section 2.3.1), *TFEB* is transcriptionally suppressed in MYC overexpressing B cells, and is thus not detected as a target of eIF5A^Hyp^ in B-cell lymphoma. Also surprising is the fact that, although the biallelic loss of *Dhps* abolishes conversion to the malignant state, *Dhps* heterozygosity provokes a more accelerated course of disease, partly due to compensatory upregulation of *Dhps* in Eμ-*Myc*;CD19-*Cre*;*Dhps^fl/+^* mice. This phenomenon is strikingly similar to the overcompensation of *Dhps* expression that is seen in the spleen, heart, and gut of *Dhps^+^*^/−^ mice versus their wildtype littermates [16].

#### 2.3.3. T Cells

Recent advances in T cell research have revolutionized medicine, offering patients new treatment options that include therapies based on antibodies that prevent immune checkpoint signaling, bispecific antibodies designed to simultaneously bind to targets and T cells, and personalized adoptive cell therapies such as chimeric antigen receptor (CAR)-T and tumor infiltrating lymphocytes (TILs) [40]. Polyamine synthesis is increased during T cell activation [41,42] and several independent studies have demonstrated the functional importance of the polyamine–hypusine circuit in T cell functions under normal and disease states.

Following activation, CD4^+^ T helper (T_H_) cells undergo a series of events, including proliferation and differentiation into distinct T cell subtypes (T_H_1, T_H_2, T_H_17, and Tregs) that have specialized functions and cytokine production. Puleston, Pearce, and colleagues have demonstrated that the polyamine–hypusine circuit controls T-cell lineage determination via epigenetic reprogramming [43]. Specifically, CD4^+^ T cells isolated from CD4-*Cre*;*Odc^fl/fl^* and CD4-*Cre*;*Dohh^fl/fl^* mice exhibit altered T_H_ subset differentiation following activation ex vivo and aberrantly express cytokines (e.g., IFN-γ) and lineage-defining transcription factors (e.g., T-bet) across T_H_ subsets. Importantly, these mice develop severe intestinal inflammation and colitis, and this is linked to increased histone acetylation marks in all T_H_ subsets of CD4-*Cre*;*Odc^fl/fl^* mice and in select T_H_ subsets of CD4-*Cre*;*Dohh^fl/fl^* mice [43]. Moreover, deletion of histone acetyltransferase (HAT) can restore proper T_H_ cell differentiation ex vivo. In accord with these findings, reduced spermidine is linked to global increases in histone acetylation marks (H3K9Ac, K14Ac, and K18Ac) in yeast *S. cerevisiae* [34].

As noted above, Type 1 diabetes (T1D) is caused by immune-mediated destruction of insulin-secreting β cells in the pancreas, and in vivo inhibition of eIF5A^Hyp^ by GC7 treatment has been shown to delay the onset of T1D in a humanized mouse model of T1D [44]. Here, inhibition of eIF5A^Hyp^ by in vivo GC7 treatment alters T_H_ subsets by reducing T_H_1 and T_H_17 cells and by enriching immune suppressive regulatory T cells (Tregs), which reduces inflammation and promotes β cell functionality in terms of insulin release and reduced ER stress [45]. Importantly, however, inhibition of eIF5A^Hyp^ does not abrogate the CD8^+^ cytotoxic T lymphocyte (CTL)-mediated destruction of β cells, indicating that simultaneous blocking of autoreactive CTLs in the islet environment is also required to prevent T1D.

Similarly to the studies of B cells noted above (see Section 2.3.1), immune senescence of CD8^+^ T cells is mediated in part by age-related reductions in autophagy, and spermidine supplementation can induce autophagy, increase antigen-specific CD8^+^ T cells, and improve the CD8^+^ T cell responses to vaccination and to infections in old mice [35]. Thus, the next question is whether eIF5A^Hyp^ mediates age-related immune senescence in this context.

eIF5A is required for the long-term survival of effector CD8^+^ T cells and eIF5A is abundantly expressed in naïve CD8^+^ T cells [29]. Upon T cell activation, however, eIF5A and DHPS are further upregulated, leading to increased levels of eIF5A^Hyp^ [29], which in turn controls proliferation, survival, and cytokine production, specifically of IFNγ. In such activated cells, eIF5A-dependent translational targets, which were identified by the proteomics analysis of the newly synthesized peptides labeled by the incorporation of 4-Azido-L-homoalanine (AHA) in *Eif5a* knockout cells, include the cell cycle regulator CDK1 and the TBET and IRF4 transcription factors that control the production of IFNγ and to a lesser extent TNF (Table 2). The study by Tan and colleagues has also provided a few additional noteworthy points. Firstly, GC7 treatment did not phenocopy the knockout cells. For example, while the *Eif5a* or *Dhps* knockout cells accumulated in G0/1 phase cells, which is consistent with our cell cycle analysis of *Dhps* knockout B-cell lymphoma cells [28], GC7-treated activated CD8^+^ T cells accumulated in S phase, indicating possible off-target effects on cell cycle progression [29]. Secondly, the proteomics analysis of nascent proteins identified 2617 downregulated proteins in GC7-treated cells compared with only 234 downregulated proteins in the knockout cells, indicating another example of the off-target effects of GC7. Finally, *Eif5A* knockout exhibited more profound phenotypes than those from *Dhps* or *Dohh* knockout CD8^+^ T cells, indicating that unmodified eIF5A retains translational activity to some extent. For example, reductions of autophagic flux are observed in *Eif5a* knockout CD8^+^ T cells but not in *Dhps*- or *Dohh*-deficient CD8^+^ T cells.

#### 2.3.4. Macrophages

Macrophages are involved in a wide variety of physiological functions, including phagocytosis and tissue repair and remodeling [46,47]. Systemic signals and locally secreted stimuli can activate macrophages into specialized phenotypes, which can be classified into M1 (classic activation) or M2 (alternative activation) macrophages. To date, four major studies have addressed the roles of eIF5A^Hyp^ in macrophage differentiation.

Loss of ornithine decarboxylase (ODC), the first and rate-limiting enzyme of polyamine biosynthesis that converts ornithine into putrescine, promotes classical M1 macrophage activation [48], and polyamine biosynthesis is activated during alternative macrophage activation [30]. Further, mitochondrial metabolism is modulated by eIF5A^Hyp^ in this context, where pharmacological inhibition of the polyamine–hypusine circuit impairs oxidative phosphorylation (OXPHOS)-dependent M2 macrophage activation while maintaining aerobic glycolysis-dependent M1 macrophage activation [30]. Proteomics analysis of mouse bone marrow-derived macrophages (BMDMs) activated by interleukin-4 (IL-4) with or without GC7 treatment identified 153 significantly altered proteins, of which ~ 40% were mitochondrial proteins, suggesting eIF5A^Hyp^ is required for proper mitochondrial function. In support of this notion, eIF5A^Hyp^-dependent targets in macrophages include succinyl-CoA synthetase (SUCLG1), succinate dehydrogenase (SDH), and methylmalonyl-CoA mutase (MCM), which are specifically required for maintenance of the TCA cycle (Table 2). Interestingly, mitochondrial targeting sequences (MTSs) in some of these mitochondrial proteins show an increased dependency on eIF5A^Hyp^.

Similarly, Nakamura and colleagues have observed elevated levels of eIF5A^Hyp^ in BMDMs activated by IL-4 but not in those treated with LPS + IFN-γ; they further demonstrated the upregulated expression of components of complexes I, II, and IV of the electron transport chain in macrophages treated with IL-4 but not in those treated with LPS + IFN-γ [49]. This suggests a possible link of eIF5A^Hyp^ to mitochondrial regulation during alternative (M2) activation of macrophages. In accord with this notion, treatment with the ODC inhibitor difluoromethylornithine (DFMO) reduces levels of eIF5A^Hyp^ in BMDMs and levels of CI, CII, and CIV proteins, and exogenous putrescine rescues DFMO-induced reductions of eIF5A^Hyp^ and these potential eIF5A^Hyp^ targets. Moreover, uptake of commensal bacterium-derived putrescine facilitates colonic epithelial cell proliferation/renewal and increases the abundance of M2 macrophages in the colon, and these effects may occur via the hypusination of eIF5A.

Myeloid cells in the mammalian gastrointestinal tract respond to inflammatory signals and to foreign antigens to clear pathogens. Two studies have evaluated the long-term consequences of eIF5A^Hyp^ loss on pathogen clearance or metabolic inflammation (meta-inflammation [50]) using myeloid lineage cells lacking *Dhps* [31,32]. Firstly, gastrointestinal pathobionts such as *Helicobacter pylori* (*H. Pylori*) and *Citrobacter rodentium* (*C. rodentium*) induce upregulation of *Dhps*, resulting in increased intracellular levels of eIF5A^Hyp^ in macrophages in the GI tract [31]. Proteomic and immunoblot analysis of BMDMs from *H. Pylori*-infected myeloid lineage-specific *Dhps* knockout (*Dhps^fl/fl^*;Lyz2-*Cre*) mice revealed the antibacterial effectors IRG1 and NOS2 and the autophagy-regulatory factor SQSTM/sequestrin/p62 as eIF5A^Hyp^-regulated proteins (Table 2). In addition, these three factors are induced in BMDMs from *C. rodentium*-infected mice, whereas levels of these proteins are reduced in the BMDMs from *C. rodentium*-infected *Dhps^fl/fl^*;Lyz2-*Cre* mice, confirming that loss of *Dhps* in macrophages results in a failed antibacterial response to these pathobionts, indicating pivotal roles of eIF5A^Hyp^ in innate immunity in the gastrointestinal mucosa.

In obesity, increased proinflammatory M1 macrophages are present in adipose tissue, causing meta-inflammation [51]. Levels of eIF5A^Hyp^ are also elevated in adipose tissue macrophages from obese mice [32]. Proteomics analysis of either M1- or M2-polarized BMDMs isolated from the *Dhps^fl/fl^*;Lyz2-*Cre* mice revealed that i) the levels of NF-κB regulators IL17RA, STK11/LKB1, TRIM13, PARP1, and IκBα are reduced following the loss of *Dhps* in BMDMs polarized under M1 conditions, and ii) 53 proteins are translationally altered in BMDMs polarized under M2 conditions yet are not clustered into any one signaling pathway. Indeed, in contrast to the studies by the first two groups above [30,49], mitochondrial OXPHOS proteins were not identified [32], which could be due to differences in the way of eIF5A^Hyp^ inhibition (myeloid lineage-specific *Dhps* deletion versus GC7 treatment and/or chronic versus acute effects of eIF5A^Hyp^ inhibition). Importantly, the transcription factor NF-κB plays a central role in M1 polarization by inducing the expression of genes encoding proinflammatory cytokines and chemokines. Further, loss of *Dhps* in macrophages (*Dhps^fl/fl^*;Lyz2-*Cre* mice) impairs translation of the transcripts encoding the proinflammatory cytokine IL-1-beta (*Il1b*) and the chemokine MIP-1a (*Ccl3*) [32]. Thus, loss of *Dhps* in myeloid cells of obese mice results in the reduced accumulation of M1 macrophages in adipose tissue, which ameliorates glucose tolerance.

#### 2.3.5. Hematopoietic Stem and Progenitor Cells (HSPCs)

Differentiation of HSPCs to the erythroid lineage is unique in that progressive mitoses lead to the generation of enucleated reticulocytes. Recent studies by Gonzalez-Menendez et al. have shown that arginine uptake and the resulting polyamine spermidine play critical roles in erythroid differentiation, and that this occurs via effects on translational control by eIF5A^Hyp^ [33]. Furthermore, the pharmacological inhibition of DHPS or depletion of DHPS by knockdown in erythroid progenitors attenuates human erythroid but not myeloid cell differentiation. Proteomics analysis of EPO (erythropoietin)-stimulated CD34^+^ cells that were treated with GC7 revealed the loss of proteins involved in mitochondrial translation, linking the translational control of mitochondrial translation apparatus by eIF5A^Hyp^ to erythroid differentiation, mitochondrial function, and reduced oxidative phosphorylation. Interestingly, although the mechanism by which eIF5A^Hyp^ facilitates translation of mitochondria proteins is not clear, mitochondrial ribosomal proteins were reduced upon loss of hypusination. Finally, the ineffective erythropoiesis manifest in haploinsufficiency of *RPS14* (ribosomal protein S14) in chromosome 5q deletions in myelodysplastic syndromes is associated with reduced eIF5A^Hyp^ levels, and *RPL11* (ribosomal protein L11)-haploinsufficiency in Diamond–Blackfan anemia is associated with CD34^+^ progenitors having reduced eIF5A^Hyp^ and RPL11 expression.

### 2.4. Other Noteworthy Topics

#### 2.4.1. Roles of DOHH

The last step of hypusination, deoxyhypusine hydroxylation, is mediated by DOHH. Despite several recent studies focusing on DHPS or eIF5A, those studies specifically targeting DOHH are limited. This is in part due to the fact that depletion of DHPS or eIF5A has shown significant phenotypes, while the depletion of DOHH exhibits much less or milder phenotypes at cellular levels. For example, the deletion of *Lia1*, the yeast ortholog of DOHH, shows no overt phenotype under normal growth conditions [52]. On the other hand, loss of *Dohh* in mice leads to embryonic lethality and germline variants in *DOHH* are associated with severe neurodevelopmental disorder in humans, indicating that DOHH also plays essential roles in development.

One interesting study by Zhang et al. has shown that oxygen levels regulate DOHH/Lia1 activity in yeast, and that this in turn controls translation of select proteins involved in oxidative phosphorylation, the oxidative stress response, and protein folding [53]. Notably, the loss of deoxyhypusine hydroxylation by the deletion of *Lia1* specifically impairs the translation of the *N*-termini (~10 amino acids) of these proteins, which relies on the interaction of the *N*-terminal nascent peptide with the peptide exit tunnel of the ribosome. Interestingly, this selective translation that is reliant on oxygen-sensing DOHH is independent of polyproline-containing motifs.

The overexpression of *EIF5A* and *DOHH* is a hallmark of many tumor types and there are modest increases in *DHPS* expression in many cancer types [28]. Furthermore, all three genes are highly elevated in human and mouse lymphomas that have *MYC* involvement. Moreover, *EIF5A*, *DHPS*, and *DOHH* are also highly expressed in glioblastoma (GBM), the most aggressive primary brain tumor in adults with a poor prognosis (a 5-year survival: ~5% [54]) [55]. Additionally, an elevated expression of DOHH has been identified as one of the molecular markers that is associated with the poor prognosis of GBM, where proteomic analysis of 84 GBM patients revealed that DOHH was highly expressed in short-term (<6 months) survivors compared with long-term survivors [56].

#### 2.4.2. Free Pools of Hypusine

The unique amino acid hypusine was first isolated from bovine brain tissue in 1971 [57] and was subsequently found in other tissues, including liver, kidney, muscle, and blood [58]. The free form of hypusine results from the proteolytic degradation of eIF5A^Hyp^ [59]. Five decades later, a recent study testing the biological activity of hypusine in C6 rat glioma cells showed that hypusine treatment reduced proliferation and its clonogenic potential without leading to apoptosis [60]. Interestingly, the treatment with hypusine resulted in the reduction of global protein synthesis by 40% and of *Eif5a* mRNA levels but not the reduction of total eIF5a or eIF5A^Hyp^ protein. Further, testing the effects of free hypusine in combination with temozolomide, the frequently used chemotherapy agent for GBM, impaired cell proliferation synergistically [60].

## 3. Conclusions and Perspectives

Great progress has been made towards understanding the roles of eIF5A^Hyp^ in various tissue, cell type, and physiological contexts, along with new methods of acutely and chronically inhibiting eIF5A^Hyp^. Despite rather modest effects on total protein synthesis, loss of eIF5A^Hyp^ is associated with many profound phenotypes. Commonly reported defects in cellular functions associated with eIF5A^Hyp^ inhibition include impairments in cell proliferation, cell cycle arrest (G1/S), reductions of autophagy, and mitochondrial dysfunction. Interestingly, eIF5A^Hyp^ translational targets are often tissue/cell context specific, even when the same cellular function is targeted and controlled by eIF5A^Hyp^ (e.g., autophagy: ATG3 in breast cancer and TFEB in B cells) (Table 2).

eIF5A^Hyp^ is also required for proper development, cell fate lineage commitment of erythroid and T cells, and proper macrophage polarization. Further, decreased levels of eIF5A^Hyp^ resulting from age-induced reductions in spermidine result in autophagy dysfunction in B cells and CD8^+^ T cells in old mice, possibly leading to immune senescence (decline of immune function with age). Taken together, eIF5A^Hyp^ is essential for regulation and maintenance of the immune system.

An almost complete loss of hypusine provokes profound phenotypes in different tissues and cell types, which is likely due to the pleiotropic effects of eIF5A^Hyp^ inhibition. Given this, it is important to assess potential systemic side effects of therapies targeting the hypusine circuit in both normal and pathophysiological conditions. Because eIF5A hypusination appears to be the sole function of DHPS and DOHH, targeting these enzymes may be a specific and attractive therapeutic strategy for translating some of these preclinical findings noted here into the clinic. Given several off-target effects of GC7, a spermidine analog, discussed above, the development of improved small molecule inhibitors of DHPS or DOHH, or agents that efficiently block eIF5A^Hyp^ function, is clearly needed. Free hypusine molecules tested in GBM may be another promising strategy and understanding the mechanism of this inhibition is another needed area of investigation.

## Figures and Tables

**Table 1:** List of transgenic mice targeting the genes in the hypusine axis.

Gene	Alleles	Target Regions	Group	References
*Dhps*	*Dhps^+/gt^*	Gt ^1^ in exon 2	Park	Nishimura et al., 2012 [14]
	*Dhps^fl/fl^*	Exon 2–7	Balabanov	Pallmann et al., 2015 [16]
	*Dhps^fl/fl^*	Exon 2–7	Mirmira	Levasseur et al., 2019 [20]
	*Dhps^N173S^*	N173S	Lutz	Donated to the Jackson lab (JAX)
*Dohh*	*Dohh^fl/fl^*	Exon 2–4	Balabanov	Sievert et al., 2014 [15]
*Eif5a*	*Eif5a* ^+/*gt*^	Gt in intron1	Park	Nishimura et al., 2012 [14]
	*Eif5a^+/K50R^*	K50R	Bachmann	Shultz et al., 2023 [21]
*Eif5a2*	*Eif5a2^fl/fl^*	Exon 2–3	Balabanov	Pallmann et al., 2015 [16]
	*Eif5a2^+/K50R^*, *Eif5a2^K50R/K50R^*	K50R	Bachmann	Shultz et al., 2023 [21]

^1^ Gt—gene trap.

**Table 2:** List of cell-specific eIF5A^Hyp^ translational targets.

Tissue/Cell Type	Mouse Model or Cell/Condition *	Primary Method	Additional Method	Major Representative Targets	Cellular Function	References
IECs ^1^	*Dhps^fl/fl^*;Vil1-*Cre*	Proteomics	IB ^2^	GSTA4, GSTM3, GSTM2, GSTP1, GSTO1, GSTM1, AL1A7, AL1B1, ALDH2	Aldehyde detoxification	Gobert et al., 2023 [22]
Pancreatic islet β cells	*Dhps^fl/fl^*;MIP1-*CreERT* on HFD ^3^	Proteomics	IB	Cyclin D2	Proliferation	Levasseur et al., 2019 [20]
PDAC ^4^	PANK1, 779E/knockdown of *EIFA*, *EIF5A2*, or both genes, GC7, or CPX treatment	IB		PEAK1	Src kinase activity	Fujimura et al., 2014 [24]
PDAC	779E cell/knockdown of *EIF5A*	Proteomics	IB	RhoA, ROCK2, TRIM29, XRN1, ZO1	Rho/ROCK signaling, cell motility	Fujimura et al., 2015 [25]
Breast cancer	MCF-7 cell/knockdown of *EIF5A*	Proteomics	IB	ATG3	Autophagosome formation	Lubas et al., 2018 [26]
B cells	Primary B cells/GC7 treatment	Proteomics	IB	TFEB	Autophagy	Zhang et al., 2019 [27]
B-cell lymphoma	Eμ-*Myc* lymphoma/knockdown of *Eif5a* or *Dhps*	RP ^5^, proteomics	IB	POLD1, E2F, PIM3, SCD1, Cyclin D3	Cell cycle, replication, proliferation	Nakanishi et al., 2023 [28]
T cells	OT-1 CD8^+^ T cells/knockout of *Eif5a* or GC7 treatment	Proteomics	FACS	CDK1, TBET, IRF4	Cytokine production	Tan et al., 2022 [29]
Macrophages	BMDMs (+IL-4) ^6^/GC7 treatment	Proteomics	IB	SUCLG1, SDH, MCM, pyruvate dehydrogenases	TCA cycle, ETC	Puleston et al., 2019 [30]
Macrophages	*Dhps^fl/fl^*;Lyz2-*Cre* BMDMs (+*H. pylori*) ^7^	Proteomics	IB	NOS2, IRG1, SQSTM	Antibacterial response, autophagy	Gobert et al., 2020 [31]
Macrophages	*Dhps^fl/fl^*; Lyz2-*Cre* BMDMs (+LPS + IFN-γ) ^8^ *Dhps^fl/fl^*; Lyz2-*Cre* BMDMs (+IL-4)	Proteomics	IB, RIP ^9^, PP ^10^	M1: IL17RA, SRK11/LKB1, TRIM13, PARP1, IκBα, CCL3, IL1b M2 ^11^	NF-κB signaling, proinflammatory signaling	Anderson-Baucum et al., 2021 [32]
HSPCs ^12^	CD34^+^ cells (+EPO)/GC7 treatment	Proteomics		Mitochondrial proteins, including mitochondrial ribosomal proteins	Mitochondria function/OXPHOS	Gonzalez-Menendez et al., 2023 [33]

* Cells/conditions used in the primary screen method are shown.^1^; IECs—intestinal epithelium cells ^2^; IB—immunoblotting ^3^; HFD—high-fat diet ^4^; PDAC—pancreatic ductal adenocarcinoma ^5^; RP—ribosome profiling ^6^; BMDMs (IL-4)—mouse bone marrow-derived macrophages (M2 polarization) ^7^; infected with *H. pylori* ^8^; BMDMs (+LPS + IFN-γ)—mouse bone marrow-derived macrophages (M1 polarization) ^9^; RIP;—RNA immunoprecipitation ^10^; PP—polysome profiling followed by qPCR ^11^. Fifty-three proteins were found in *Dhps* knockout macrophages, and KEGG analysis showed no clustering. Also, note that eIF5A^Hyp^ levels were not altered under M2 macrophage conditions. ^12^; HSPCs—hematopoietic stem and progenitor cells.

## Data Availability

Not applicable.
